# The experience of polyethylene glycol (PEG) bowel preparation in adolescents undergoing colonoscopy

**DOI:** 10.1186/s13104-020-05011-7

**Published:** 2020-03-26

**Authors:** Karin Örmon, Ann-Cathrine Bramhagen, Charlotta Sunnqvist, Vedrana Vejzovic

**Affiliations:** Department of Care Science, Faculty of Health and Society, Malmö University, 20506 Malmö, Sweden

**Keywords:** Adolescents, Pediatric colonoscopy, Polyethylene glycol

## Abstract

**Objective:**

The aim of this study was to describe the experience of polyethylene glycol (PEG) bowel preparation in adolescents undergoing colonoscopy.

**Results:**

32 adolescents, 10–18 years of age self-reported a minimum of complications 1 week after colonoscopy when PEG was used for bowel preparation. 17 adolescents, 10–18 years were also interviewed about bowel preparation with PEG. Using qualitative content analysis, two categories were extracted from the data: “Being decisive makes it manageable” and “Be prepared for a horrible experience.” The adolescents reported PEG intake difficulty; the intake was, however, manageable if they received appropriate information.

## Introduction

Colonoscopy is the gold standard for the diagnosis of pediatric inflammatory bowel disease, IBD, where the rectum and the lower bowel are examined for abnormalities and disease [1]. Adolescents with symptoms of IBD undergo an initial evaluation for IBD, and must often be subjected to a series of different diagnostic tests, including colonoscopy with biopsies. These adolescents have frequently endured long periods of gastrointestinal (GI) symptoms, such as abdominal pain, diarrhea, weight loss, GI bleeding, growth failure, and anemia [1], which can have a negative impact on adolescents’ daily life [2–5]. GI symptoms, in combination with the large quantities of laxative fluid needed for bowel cleansing during the pre-colonoscopy procedure, may be the reason that bowel cleansing prior to colonoscopy has been found to be difficult for both the adult patient [6] and children [7, 8]. According to the literature, about 25 percent of all IBD cases are diagnosed during adolescence [9], which means that the group that must undergo a colonoscopy is not insignificant. The pre-colonoscopy procedure involves hospitalization, bowel cleansing including diet and fasting, anesthesia, and blood samples, and it is not surprising that the pediatric patients experienced it as difficult.

Polyethylene glycol (PEG) is generally recommended as a standard laxative for pediatric pre-colonoscopy due to its bowel-cleansing efficacy [10], but previous research has shown that bowel cleansing with PEG is experienced as a difficult part of the pre-colonoscopy procedure [7]. To better understand how pediatric patients experience the procedure and to find solutions that can facilitate it for those patients, we need more knowledge from the patients’ perspective.

Therefore, the aim of this study was to describe the experience of polyethylene glycol (PEG) bowel preparation in adolescents undergoing colonoscopy.

## Main text

### Methods

**Fig. 1:**
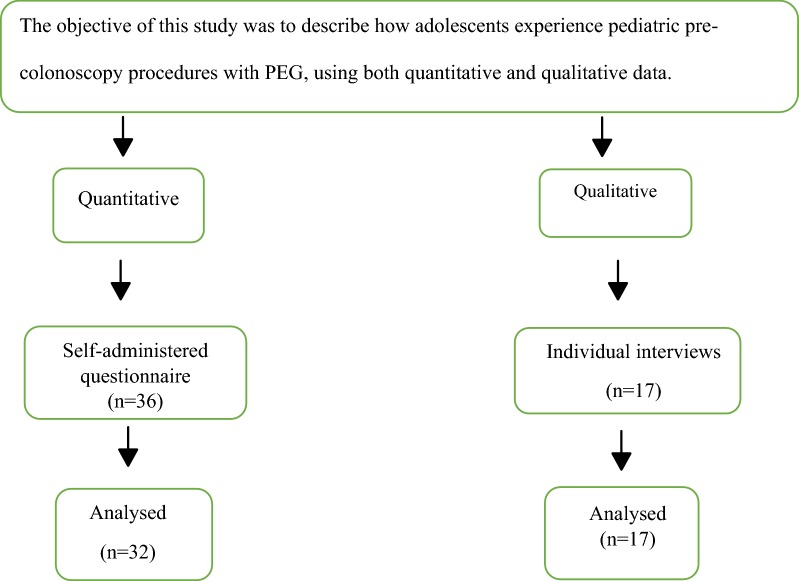
Flowchart of method and participants

This study was based on both quantitative and qualitative data as a third part of a large study (Fig. 1) [7, 11].

#### Setting and participants

The data for this study were collected in 2012 (17 interviews) and 2015 (32 self-reported questionnaires) and a summary of the demographic data is presented in Table 1. The recruitment of patients (10–18 years of age) was conducted at a university hospital in the south of Sweden. The inclusion criteria were: being an adolescent with suspected IBD who had undergone an elective colonoscopy where PEG was used for bowel cleansing.

**Table 1:** The baseline characteristics of the included patients

Quantitative	Qualitative
Participants 32	17
Sex N (%)	
Boys 17 (54.4)	5 (29, 42)
Girls 15 (46.6)	12 (70, 58)
Age 10–18	10–17

##### Bowel preparation

All adolescents included in the current study were inpatients during the bowel preparation prior to colonoscopy. They received a weight-adjusted dosage of PEG 3350 with electrolytes (BioPhausia, Stockholm, Sweden): 25–35 mL/kg body weight per hour until clear intestinal fluid was obtained, either orally or by nasogastric tube. Some received PEG by nasogastric tube when they were unable to manage an oral intake of the prescribed dose. Participants were provided with written dietary instructions; these instructions allowed for low residue food for 4 days before the procedure and recommended no solid food intake for at least 24 h before the colonoscopy, and only clear liquid was recommended during the bowel preparation.

#### Data collection

The quantitative data consisted of self-reported questionnaires with a focus on how the adolescents felt 1 week after bowel cleansing with PEG (Table 2). The adolescents were asked to answer 10 questions. They ranked the tolerability of the laxative after 1 week, using a 4-point scale: “not at all,” “a little,” “a lot,” and “very much.”

The qualitative data were collected through individual interviews. Each interview was based on one question: “How would you describe bowel cleansing with PEG to your peers who need to undergo the same procedure?” The interviews were recorded and transcribed verbatim. The Regional Ethical Review Board in Lund granted ethical approval (Ref. No. 2011/155; No. 2012/464).

#### Data analysis

Descriptive statistics was used for the quantitative analysis with frequency distribution in number, percent, and median. The interviews were analyzed with content analysis [12]. Open coding was used to create categories of text in the results, which were discussed among the authors.

### Results

#### Tolerability 1 week after bowel preparation

The potential population for the self-reported questionnaires was 36 adolescents 1 week after they had undergone first-time colonoscopy. The total response rate was 88.9% (32/36) and the mean age of the adolescents was 15.3 (SD = ± 1.9). The results are presented in Table 2. The adolescents reported a bloated stomach (8/32) and stomach ache (10/32). Moreover, eight adolescents reported not being able to sleep 1 week after bowel cleansing with PEG.

**Table 2:** Self-reported complications 1 week after PEG intake (n = 32)

	Not at all	A little	Much	Very much
	n (%)	n (%)	n (%)	n (%)
Have I had a bloated stomach	8 (25.0)	16 (50.0)	5 (15.6)	3 (9.4)
Have I had feces	4 (12.5)	20 (62.5)	5(15.6)	3 (9.4)
Have I had stomach ache	9 (28.1)	13 (40.6)	7 (21.9)	3 (9.4)
Have I felt like vomiting	17 (53.1)	12 (37.5)	3 (9.4)	0 (0.0)
Have I had headache	21 (65.6)	10 (31.3)	1 (3.1)	0 (0.0)
Have I felt worry	23 (71.9)	7 (21.9)	2 (6.3)	0 (0.0)
Have I had difficulty sleeping	24 (75.0)	7 (21.9)	1 (3.1)	0 (0.0)
Have I felt sad	22 (68.8)	10 (31.3)	0 (0.0)	0 (0.0)
Have I been able to talk about what				
I have gone through	1 (3.1)	9 (28.1)	10 (31.3)	12 (37.5)
Have I had others problems	24 (75.0)	7 (21.9)	0 (0.0)	1 (3.1)

#### Adolescents’ experiences 1 week after colonoscopy

The result of the qualitative data analysis can be presented as two categories: “Be prepared for a horrible experience” and “Being decisive makes it manageable.” The adolescents would, they said, inform their peers that there are two options for the intake of PEG for bowel preparation: they could do it orally or by means of a nasogastric tube. They reflected that choosing an alternative was difficult because both options were considered unpleasant; the oral one because of the bad taste and the large volume, and the tube because of the discomfort of the insertion procedure. They would also describe to their peers, how anxiety and discomfort were evident during the procedure, but that, in retrospect, a week later, the procedure did not feel so difficult.

Furthermore, the adolescents’ narratives showed that they would inform their friends that drinking PEG was awful and almost unbearable.

*It’s difficult to describe to friends, but even so it’s important to know that it’s difficult to drink this volume* [3]

#### Be prepared for a horrible experience

The need to be prepared for the fact that drinking PEG is awful and difficult was thus something that the adolescents would tell their peers about. Information about nausea, and about the amount of liquid and how difficult it was to manage, would also be shared with peers who would need to undergo the pre-colonoscopy bowel preparation.

I’d like to tell them that this laxative… it’s probably the worst part. What’s worst is having to stand drinking it, especially the last amount. It makes one so nauseous, that’s something one has to prepare for [10].

Drinking a few glasses of the liquid would be manageable, they claimed, but drinking more was difficult and even “impossible.” Especially the last glasses of PEG were almost unmanageable. The adolescents said that they would inform their friends that a tube might be the best option.

*I recommend the tube; the first three glasses are okay, but then it’s Stop!* [7]

#### Being decisive makes it manageable

Some of the narratives illuminated the importance of maintaining strategies to be able to drink PEG if they chose the oral intake. In those narratives, the adolescents concluded that drinking PEG was manageable and something they just had to do.

*Just drink the two liters and get on with it, just do it* [6]

This state of mind would help their friends to manage the procedure, they said. In this context it is important to point out that the healthcare staff were perceived as experienced and kind, and that the adolescents found it helpful to ask the staff about things that they were worried or concerned about. Even when being scared and stressed due to the PEG intake, the adolescents knew that everything would be fine and safe, and they asserted that being calm and focused was preferable and made the procedure bearable.

### Discussion

The result from the present study suggests that despite difficulties with the PEG intake adolescents manage to drink the requested amount, with a minimum of physical complications reported 1 week after the procedure. Previous results have shown complications, such as sleeping problems and worrying in connection with bowel preparation with PEG [7, 8]. In this study, however, more than 90 percent of the adolescents reported no/little complication with vomiting, headache, sleeping, and worrying, 1 week after the bowel preparation. This is important knowledge, because of the recommendation for pediatric bowel preparation with PEG [10, 13]. The adolescents also, 1 week afterwards, described the bowel preparation as easier than they had imagined. It is difficult to determine, on the basis of our quantitative results, whether these responses reflect merely the bowel preparation or the entire procedure. However, judging by the adolescents’ narratives, this part of the procedure can clearly be seen as difficult and important to recount to others who will undergo the same procedure.

The adolescents in this study also reported that they could talk much/very much (68.8%) about the procedure 1 week after the colonoscopy, even though research has described an unwillingness to talk about the procedure before and during pre-colonoscopy [7]. This could be important to bear in mind for healthcare staff when they take care of adolescents who need to undergo colonoscopy repeatedly.

The result of the qualitative analysis showed that adolescents need and wish to be informed about the experiences of others, including the difficulties. They need to understand the whole procedure and their options, before they can make decisions and manage the procedure. If healthcare staff informed adolescents beforehand and in an understanding way regarding alternative ways of undergoing the PEG bowel preparation, the procedure would probably be more manageable for the adolescents. By being presented with different strategies for the PEG intake, the adolescents would have alternatives as well as getting guidance and thereby hopefully a feeling of control and of being listened to, which would be in accordance with both child-centered care and recommendations [13].

### Conclusion

The results indicate a need for an individual, customized PEG bowel preparation, where the requirements and needs of the adolescent are in focus. The result of the study also pinpoints that adolescents ought to be allowed to choose their own PEG bowel preparation.

## Limitations

This study has several limitations. The relatively small size restricted the possibility to make strong inferences. Another limitation is that we are not able to report if the use of the nasogastric tube was favored among the younger or older adolescents. However, the aim was not to describe whether an oral intake or the nasogastric tube was preferable. The qualitative component of the analysis was chosen to strengthen and support key quantitative findings. The present study was conducted in a single setting, thereby limiting generalizability. Nevertheless, the study site was a primary pre-colonoscopy procedure referral clinic and the adolescents in this study are likely a representative group.

## Data Availability

The datasets generated and/or analyzed during the current study are not publicly available due to ethical considerations, but are available from the corresponding author on reasonable request.

## Abbreviation

PEG: Polyethylene glycol

## References

[1] ESPGHAN, European Society for Paediatric Gastroenterology, Hepatology and Nutrition, IBD Working group (2005). Inflammatory bowel disease in children and adolescents: recommendations for diagnosis—the Porto criteria. J Pediatr Gastroenterol Nutr.

[2] Mamula P, Markowitz JE, Baldassano RN (2003). Inflammatory bowel disease in early childhood and adolescence: special considerations. Gastroenterol Clin N Am.

[3] Mackner LM, Bickmeier RM, Crandall WV (2012). Academic achievement, attendance, and school-related quality of life in pediatric inflammatory bowel disease. J Dev Behav Pediatr.

[4] Thakkar K, Halub JL, Gilger MA (2016). Quality indicators for pediatric colonoscopy: results from a multicenter consortium. Gastrointest Endosc.

[5] Rabizadeh S, Dubinsky M (2013). Update in pediatric inflammatory bowel disease. Rheum Dis Clin N Am.

[6] McLachlan SA, Clements A, Austoker J (2012). Patients’ experiences and reported barriers to colonoscopy in the screening context—a systematic review of the literature. Patient Educ Couns.

[7] Vejzovic V, Wennick A, Idvall E (2015). A private affair: children’s experiences. J Clin Nurs.

[8] Di Nardo G, Aloi M, Cucchiara S, Spada C, Hassan C, Civitelli F, Nuti F, Ziparo C, Pession A, Lima M, La Torre G, Oliva S (2014). Bowel preparations for colonoscopy: an RCT. Pediatrics.

[9] Kappelman MD, Rifas-Shiman SL, Kleinman K (2007). The prevalence and geographic distribution of Crohn’s disease and ulcerative colitis in the United States. Clin Gastroenterol Hepatol.

[10] Mathus-Vliegen E, Pellisé M, Heresbach D (2013). Consensus guidelines for the use of bowel preparation prior to colonic diagnostic procedures: colonoscopy and small bowel video capsule endoscopy. Curr Med Res Opin.

[11] Vejzovic V, Wennick A, Idvall E (2016). Polyethylene glycol- or sodium picosulphate-based laxatives before colonoscopy in children. J Gastroenterol Nutr.

[12] Burnard P (1996). Teaching the analysis of textual data: an experiential approach. Nurse Educ Today.

[13] Pall H, Zacur GM, Kramer RE (2014). Bowel preparation for pediatric colonoscopy: report of the NASPGHAN Endoscopy and Procedures Committee. J Pediatr Gastroenterol Nutr.

